# Circular instead of hierarchical: methodological principles for the evaluation of complex interventions

**DOI:** 10.1186/1471-2288-6-29

**Published:** 2006-06-24

**Authors:** Harald Walach, Torkel Falkenberg, Vinjar Fønnebø, George Lewith, Wayne B Jonas

**Affiliations:** 1 University of Northampton & Samueli Institute – European Office, School of Social Sciences, Park Campus, Northampton NN2 7AL, UK; 2 Karolinska Institutet, Center for Studies of Complementary Medicine, Department of Public Health Sciences, Division of International Health (IHCAR) and Department of Nursing, Stockholm, Sweden; 3 National Research Center in Complementary and Alternative Medicine, University of Tromsø, Tromsø, Norway; 4 University of Southampton, Department of General Practice, Southampton, UK; 5 Samueli Institute, Alexandria VA, USA

## Abstract

**Background:**

The reasoning behind evaluating medical interventions is that a hierarchy of methods exists which successively produce improved and therefore more rigorous evidence based medicine upon which to make clinical decisions. At the foundation of this hierarchy are case studies, retrospective and prospective case series, followed by cohort studies with historical and concomitant non-randomized controls. Open-label randomized controlled studies (RCTs), and finally blinded, placebo-controlled RCTs, which offer most internal validity are considered the most reliable evidence. Rigorous RCTs remove bias. Evidence from RCTs forms the basis of meta-analyses and systematic reviews. This hierarchy, founded on a pharmacological model of therapy, is generalized to other interventions which may be complex and non-pharmacological (healing, acupuncture and surgery).

**Discussion:**

The hierarchical model is valid for limited questions of efficacy, for instance for regulatory purposes and newly devised products and pharmacological preparations. It is inadequate for the evaluation of complex interventions such as physiotherapy, surgery and complementary and alternative medicine (CAM). This has to do with the essential tension between internal validity (rigor and the removal of bias) and external validity (generalizability).

**Summary:**

Instead of an Evidence Hierarchy, we propose a Circular Model. This would imply a multiplicity of methods, using different designs, counterbalancing their individual strengths and weaknesses to arrive at pragmatic but equally rigorous evidence which would provide significant assistance in clinical and health systems innovation. Such evidence would better inform national health care technology assessment agencies and promote evidence based health reform.

## Background

### The hierarchical view of evaluation of medical interventions

Evidence Based Medicine (EBM) has installed a canon of methods that are central to the methodological reasoning for evaluating medical interventions [1]. While originally developed for the evaluation of new pharmacological products [2,3], it is also applied to whole systems intervention approaches like nursing and psychotherapy, as well as the more complex interventions of Complementary and Alternative Medicine (CAM). EBM's main tool is the randomized controlled trial (RCT). Its essential principle is random assignment of a sufficiently large number of carefully selected patients to experimental and control groups, thereby evenly distributing known and unknown confounding variables. Changes in outcome can thus be attributed to the intervention(s).

A hierarchy of methods has been described and utilized by health technology assessment (HTA) agencies, with case series, cohort studies with historical controls, non-randomized controlled studies being of lower value and having less methodological rigor than prospective RCTs. Only RCTs are considered for inclusion in many meta-analyses and systematic reviews. These form the theoretical, if often abused and misunderstood, basis for EBM and the clinical decision making process.

We would like to argue for a broader, circular view that illustrates the equivalence of research methods in non-pharmacological interventions. More specifically we will argue that there is no such thing as a single inherently ideal methodology. There are different methods to answer different questions, all of which come together in a mosaic [4] or evidence house [5]. A poorly designed and badly implemented RCT is, as a rule, less valuable than well conducted studies using other designs, and sometimes even non-randomized studies can produce more reliable and useful information than a well conducted randomized study.

## Discussion

### Assumptions and problems of the hierarchical view

The hierarchical view makes some important assumptions which are not universally valid but rarely debated (Table 1) [6,7]. All of these assumptions are problematic and sometimes false in complex interventions. They are useful for the evaluation of new pharmacological agents but even in that situation often only some of the assumptions are met.

**Table 1:** Assumptions made in conducting randomized controlled trials

Equipoise	Patient and provider do not have a preference for a treatment
Lack of knowledge	It is truly unknown which of two alternatives is "better" and there is insufficient evidence about treatment effects from other sources
Preference for specificity	Only specific effects attributable to the intervention are therapeutically valid
Context independence	There is a "true" magnitude of efficacy, or a stable effect size independent of context
Ecological and external validity knowable	The knowledge about a therapeutic effect extracted from an RCT is readily transferable into clinical practice, if exclusion and inclusion criteria of the trial match the characteristics of a given patient

#### Problems with the assumptions

##### 1. Preference and clinical equipoise

Equipoise is traditionally the most important precondition for conducting RCTs. It means that there is no preference based on systematic knowledge for a treatment over an alternative or no treatment. Clinical equipoise is considered most important. It refers to the notion that there is honest disagreement about the optimal treatment among the medical community or between important sectors of the community. Equipoise is normally fulfilled with new procedures or pharmacological agents entering phase III studies. The RCT was introduced in exactly this context initially [2,3,8,9]. There are many practices in medicine which do not follow the rationale of a pharmacological intervention or which are more complex. Nursing and caring systems, traditional healing systems, CAM, life-style and psychological interventions, as well as surgery and rehabilitation are only some examples of complex treatments. In these interventions a whole array of therapeutically active elements may be operating simultaneously and synergistically. It is therefore impossible to imply a pre-trial equipoise. This is usually the case for doctors who have undergone considerable training in specialized disciplines which themselves are founded on their own bodies of evidence. This lack of equipoise is one of the main obstacles to a systematic evaluation of surgical interventions with RCTs [10].

##### 2. Knowledge

This has to do with the influence of a large body of historical unsystematic experience within the surgical or CAM context and elsewhere. The body of historical and non-systematic experience with surgery or CAM is not considered a sufficient scientific argument for efficacy but also does not preclude systematic research. It does, however, alter clinical equipoise. Therefore, patients willing to be enrolled in a surgical or CAM study may be different from those actively seeking out such treatments. If belief and positive expectations are important factors in enhancing treatment effects or even generating the preconditions for such treatment effects, then outcomes from trials where patient preference or treatment expectations are not considered are unrealistic estimators for the effects likely to occur in uncontrolled practice. Therefore failure to find an effect in a randomized trial cannot necessarily be taken as an indication of ineffectiveness. As a consequence, the outcomes gathered from rigorous and methodologically sound trials may not be generalizable to users within the community. Preliminary evidence from unsystematic experience and patient or provider preferences are strongly linked. The "stronger" and "older" the experience, the more embedded in our culture, the more it may be leading to patient and provider preferences thus altering equipoise, expectation and outcome.

##### 3. Specificity

**Figure 1:**
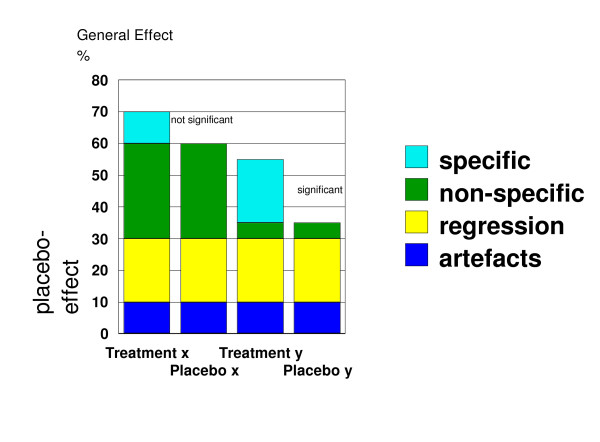
Illustration of the Efficacy Paradox. Treatment x can have a larger overall effect than treatment y, although only treatment y shows a sizeable and significant specific treatment effect; specific = specific component of treatment; non-specific = non-specific component of treatment; regression = regression to the mean, natural regression of the disease; artefacts = measurement artefacts that mimic therapeutic effects; non-specific effects, artefacts, and regression comprise the placebo effect in RCTs.

One important and rarely discussed assumptions for RCTs, especially placebo-controlled RCTs is specificity. It refers to the assumption that the only worthwhile effects are attributable to an understandable mechanism that can be clearly ascribed to a specific component of an intervention. The presupposition that only specific effects are valuable is untrue, particularly from the patient's perception. It leads to what has been called the efficacy paradox [11]. The efficacy paradox can come into play whenever complex interventions are tested against a control procedure and the control procedure implies some form of complex intervention. It can even be important in seemingly simple placebo controlled trials. This is illustrated in Fig. 1.

Consider two treatments, x and y, both tested in two controlled RCTs for efficacy. Suppose that treatment x has, overall, 70% effect, while treatment y has 55% effect in the same disease. Moreover, let us suppose that treatment x does not have a significant effect over control, or placebo x, while treatment y does. This is, because treatment y has a stronger specific effect than treatment x. Although this is, at least in principle, a matter of statistical power, let us suppose that the specific effects of treatment x are so small that they have escaped detection so far, while the general effects – specific and non-specific together – of treatment x are powerful. This leads to the efficacy paradox: A treatment that is efficacious – treatment y – can be less effective than a treatment whose efficacy has not been shown statistically different from control treatment(s) – treatment x. Research has focused only on the *difference *between the treatment condition and the control in an attempt to isolate the magnitude of the specific components of treatment, thereby neglecting the overall treatment effect. It is the latter which is most interesting to patients [12-15]. It might even be the case that the full therapeutic benefit can only be achieved in a setting that does not attempt to isolate any part of the effect, and hence trials designed to estimate the specific component of a treatment effect will fail to evaluate the full therapeutic benefit. Since placebo controlled RCTs are designed to isolate specific components they may be contraindicated in situations where the specific effects are likely to be small but the whole treatment effect large due to a complex interaction of specific and nonspecific effects [15-19].

What has been described above as a theoretical scenario has actually been empirically demonstrated meanwhile: The GERAC study (German acupuncture trial), the hitherto largest acupuncture trial, tested real acupuncture versus minimal acupuncture as a control procedure, versus pharmacological prophylaxis in migraine patients as an active treatment. This trial showed no difference between acupuncture and control, thus "proving" the "inefficacy" of acupuncture. However it also demonstrated that conventional pharmacological prophylaxis, normally considered efficacious, was not only not different from the acupuncture control, but in some secondary parameters and analyses even significantly worse than the supposedly ineffective acupuncture procedure thus illustrating the efficacy paradox [20]!

The paradox is obvious and runs thus: 1. Pharmacological prevention of migraine is considered efficacious after decades of research. 2. Sham acupuncture is not considered efficacious. In fact, it was included as a control condition. 3. The efficacy of acupuncture was contested. Hence a trial should either show superiority of the already proven intervention, pharmacological prevention, over the control condition, and equality of acupuncture with this efficacious standard treatment. The conclusion would then be: acupuncture is effective. Or else pharmacological prevention should show superiority over sham acupuncture and acupuncture, thereby disproving the efficacy of acupuncture (and sham acupuncture by default). As it happens, the conclusion can now only be: none of the interventions is effective, as none is really significantly different from the control. Hence a known effective intervention, pharmacological prevention, is rendered ineffective by the strong non-specific effect shown in the sham acupuncture (and acupuncture) group, because the logic of efficacy testing is focusing only on the difference. Clearly, this is a paradoxical and somewhat silly conclusion, but one that has to be accepted, if one wishes to live by the standards of clinical trial testing.

##### 4. Context dependence

Evidence is accumulating that the current assumptions about independence of context and setting are wrong: In two very similar trials of paracetamol, one against placebo, one against naproxen in postpartum pain, paracetamol had twice the effect in alleviating pain when subjects were expecting active treatment than when tested against placebo [21-23]. The expectancy of patients does modulate therapeutic effects so that pharmacological and psychological components are inseparable [24,25]. Naproxen under trial conditions has been reported to be significantly more effective than naproxen under normal bedside conditions, and in addition, placebo under trial conditions was more effective than naproxen under normal conditions [26]. A meta-analysis of the effects of interactions between medications and context effects found that pharmacological effects can sometimes be changed dramatically as a consequence of contextual therapeutic messages and beliefs. Pharmacological effects are *not *a stable quantity [27]. Context can be kept constant in a trial to determine efficacy and can be modulated through a variety of factors in the clinical setting, such as the belief of providers and patients [28], attitude and demeanor of the doctor [29,30], enthusiasm for the delivery of the intervention [31,32], cultural contexts and concomitant suggestions regarding diet and health [33,34]. These contextual effects can be so strong and variable that they completely overshadow the pharmacological effects of selective serotonin re-uptake inhibitors (SSRIs) [35]. Expectancy of patients seems a key factor [36,37] that has been shown to modulate therapeutic effects of anti-emetic treatments in chemotherapy [38-42] and of massage in low back pain [43,44]. Taken together these results suggest that the assumption of a "true" magnitude of a therapeutic effect, independent of context, is a very flawed construct.

##### 5. Ecological and external validity

The last assumption posits that internal validity is not only a necessary but also a sufficient condition for external validity or generalizability. An important question not answered by an RCT is that of clinical applicability: Are the effect sizes observed in an RCT reduplicated in an open clinical study? RCTs are only externally valid for the type of patients included in the trial. For an observation to be generalizable, the proportion of patients accrued for the trial must be comparatively large and representative of the condition in the community. This condition is frequently not met [7]. Depending on the intervention and the disease, patients enrolled in trials may be different from patients in clinical practice [6,45-47], primarily because selection criteria in clinical trials often do not properly reflect clinical practice [10,48,49] Selection bias could occur because the willingness to participate in a trial may be associated with certain types of patient characteristics [50]. Such a selection bias could lead to an overestimation of effects [51] if participants in trials are more positive towards the interventions than non-participants [52,53] The assumption that results of RCTs are generalizable to clinical practice is commonly made, rarely tested, and if empirically studied, often not warranted [54]. One solution proposed to solve this dilemma is large multicentre trials with thousands of participants and very few inclusion criteria [55]. However, they do not seem to be more reliable in estimating effects [56,57] than smaller studies, while at the same time they are costly and complicated. Furthermore, even mega-trials cannot test for the effect of free choice of a therapeutic method on health outcome.

If we take into account the context dependence of therapeutic effects, then it is clear that each study creates its own little universe of applicability which in the best case is an abstraction and in the worst case a distortion of the real world of clinical practice. Ecological validity is hampered the more the experimental control alters the context of clinical delivery, patient choice, and patient eligibility compared to normal practice. Thus, experimental control, while enhancing internal validity, jeopardizes external and ecological validity by default.

### The circular model of evaluation

**Figure 2:**
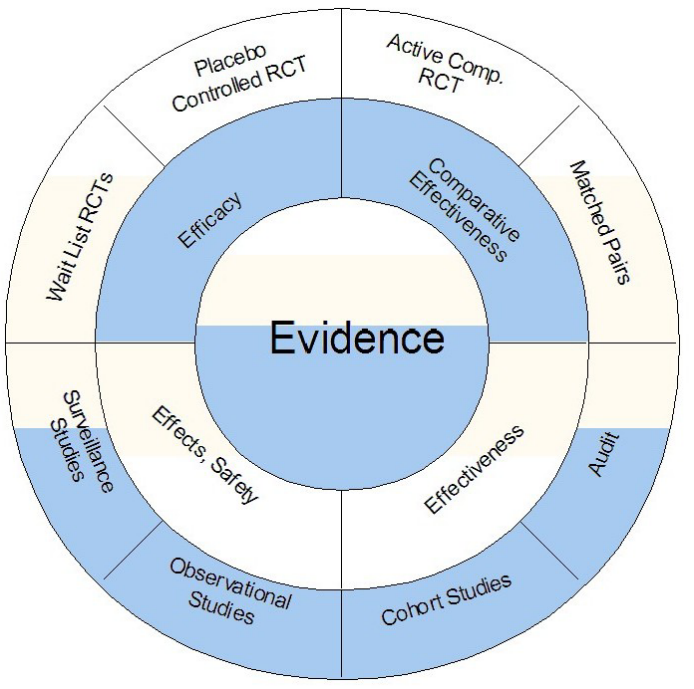
Circle of methods. Experimental methods that test specifically for efficacy (upper half of the circle) have to be complemented by observational, non-experimental methods (lower half of the circle) that are more descriptive in nature and describe real-life effects and applicability. The latter can range from retrospective audit studies, prospective case series to one armed to multiple armed cohort studies. Matched pairs studies can be conducted as experimental studies, by forming first pairs and then randomizing them, or as quasi-experimental studies by forming pairs from naturally occurring cohorts according to matching criteria. Shading indicates the complementarity of experimental and quasi-experimental methods, of internal and external validity.

The alternative to the hierarchical model is a circular one. It is derived from the experience and history of evaluation methodology in the social sciences [58-62], which has reached the consensus that only a multiplicity of methods, which are used in a complementary fashion will eventually give a realistic estimate of the effectiveness and safety of an intervention. Every research method has strengths and weaknesses which cannot be resolved within that method itself. Therefore, triangulating a result achieved with one method by replicating it with other methods may provide a more powerful and comprehensive approach to EBM when compared to the prevailing RCT approach. Rather than postulating a single "best method" this view acknowledges that there are optimal methods for answering specific questions, and that a composite of all methods constitutes best scientific evidence (Fig. 2).

The important point is not whether a study is randomized or not, but whether it uses a method well suited to answer a question and whether it implements this method with optimal scientific rigor. Figure 2 illustrates this situation: methods that are high in internal validity, such as placebo controlled RCTs or active comparator RCTs tend to be lower in external validity [63]. Thus their results need to be balanced by large and long term observational studies which document the use, safety and effectiveness of the intervention in clinical practice [64,65]. In order to assess whether an intervention has the same effect in a relevant clinical population as it does in an RCT, comparative studies in pragmatically selected cohorts are essential. If randomization proves difficult or impossible, such studies may be the only ones possible. There is some evidence that cohort studies produce effect size estimations comparable to RCTs, if conducted properly [66-68]. However, we must address the issue of variability and divergence in non-randomized studies and how this should be managed [69]. If it is enough to document effects as different from the natural course of the disease, a waiting list controlled RCT is an option. Since the intervention can be studied in its natural setting without any distortion through blinding or other restrictions, results are frequently more representative of what happens in clinical practice. Waiting list controls of up to six months are feasible in our experience depending on the condition [70]. In some cases even retrospective audits of large, well documented data sets, or better prospective documentations of pragmatically treated cohorts might give useful information about effectiveness. Single-group observational studies, in certain circumstances and with large numbers, can also yield important information [71]. If the intervention is a novel pharmacological agent, regulatory demands request that efficacy is established first, subsequent to phase 1 and phase 2 trials.

Finally, broad applicability, acceptability and a complete safety profile is established in large single-group observational trials. The previous sequence is a typical example of the steps necessary to test newly developed interventions for efficacy, applicability and safety. It has been observed that with already established interventions, such as with some CAM procedures which have a long tradition, e.g. acupuncture or homeopathy, and also with well established but little researched complex interventions such as surgical or rehabilitation procedures, the evaluation process is reversed [72]: Here, one wants to know about general clinical effectiveness and safety first. Only if this is established, are other studies warranted that probe specific efficacy, and subsequently we then develop our understanding of the underlying mechanisms of action. There are many treatments within clinical practice, where general effectiveness was established first, and specific efficacy or mechanisms of actions were discovered only later on, such as the use of aspirin, penicillin or the Western model of acupuncture. In cases where treatments have been in use for some time, the rational evaluation method starts at the non-experimental side of the circle. Thus, effect sizes will have to be established not only with the one preferred method, the RCT, but with different approaches. If convergence of effect size estimates is reached through this strategy, one can be reasonably sure about the evidence of effectiveness. If different methods have produced different results and effect size estimates, unknown moderator variables and confounding may be present. These could be the selective effectiveness of an intervention for certain subgroups. As in meta-analysis, where significant variation of effect size estimates is taken as an indicator of inhomogeneity, a lack of convergence of effect sizes in a circular strategy would be taken as an indicator of moderating influences which have to be explored.

Gabbay and Le May [1] found in their ethnographic study of decision-making in general practice that clinical decision-making is based on a combination of evidence-based medicine including systematic reviews and meta-analyses, clinical experience, individual patient need, patient and practitioner preference and peer group advice. This empirical finding supports our argument by a description of how clinicians actually come to a decision: The pyramidal hierarchy of conventional evidence-based medicine is rarely the only basis upon which clinical decisions are based in real life, primarily because such process-driven management systems almost always fail to take into account our individual nature, personal values and preferences of patients. Thus, decision making in real-life is actually much more circular than the prescriptive hierarchy of EBM would have us believe [1].

For example, many patients recover because of complex, synergistic or idiosyncratic reasons that cannot be isolated in controlled environments. The best evidence in such cases is observational data from specific clinical practices that estimates the likelihood of a patient's recovery in that practice setting. In other cases the most important information may be a highly subjective judgment about life quality. These very personal experiences of illness can only be captured with qualitative research, making this the best evidence under such circumstances. Sometimes the best evidence comes from laboratory studies. Data on the metabolic interaction of anti-virals with the herb Hypericum is crucial to the management of HIV patients [73]. Controlled trials cannot usually isolate such interactions and other surveillance methods are required. By conceptualizing evidence as circular we can highlight the fact that sometimes the "best" evidence may not be attributional, objective, additive or even clinical [5].

A circle has no preferred orientation. It might be a more fitting image for clinical research by balancing the weaknesses of one method with strengths of another. A hierarchy of methods emphasizes internal validity and experimental evidence, promoting them to a higher priority than external validity. Rather than constructing two opposing methodological approaches, one should strive for complementarity. We suggest that evidence-based medicine and HTA agencies should confront the reality of this situation in a formal manner and begin to develop a consensus-based approach that takes the evidence-based hierarchy into account, but at the same time is not ruled solely by it. Circularity, with the attendant flexibility for individualization, could provide the image describing the delicate interaction between patient and practitioner with systematic reviews, RCTs, qualitative reviews, safety, cost and individual clinical experience, all being important and recognized elements of each individualized decision-making process. Their specific importance may vary according to the individual strength of the evidence, the risks involved and the condition being treated. It might be possible for databases such as the Cochrane database to include not only issues of safety, efficacy and cost, but also evidence from patient preference and increasingly from qualitative work. The whole essence of circularity is its ability to see the whole problem within a patient-centered and human therapeutic perspective, allowing rigorous evidence, individualized decision-making at the clinical interface. We believe this is how both doctors and patients make clinical decision in the 'real world and consequently we believe our research processes should reflect this reality.

## Summary

We have argued that the widely held view of research methods forming a hierarchy is at best a simplification, at worst mistaken. Internal validity has to be balanced by external validity, and this can rarely be achieved with one single research method such as the RCT, but involves other strategies such as outcomes and cohort studies. A circular and integrative view then develops which sees research methods as particular pathways for different questions. All answers combined yield scientific evidence. Methods, then, should be viewed not in terms of a hierarchy of intrinsic worth but as valuable only relative to the question asked. To answer the question of efficacy and effectiveness, we need to triangulate different methods to achieve homogeneity. If this cannot be reached, then moderator or confounding influences must be investigated. These could be methodological in nature (bias), or systematic due to differential effectiveness and context. Such views will change the rationale of medical decision making by bringing patients, researchers and decision makers together to develop a patient-centered evidence-based consensus that will inform clinical decision making and health care reform.

## Competing interests

The author(s) declare that they have no competing interests.

## Authors' contributions

HW: Conceived the general idea, wrote the first drafts and outline of the argument and participated in redrafting and rewriting.

TF: Critically read the manuscript and contributed clinical-practical information

VF: Critically read the manuscript, added structure and argumentative content

GL: Critically read and redrafted the original manuscript, adding information about the ethnographic aspects

WBJ: Was a contributor at an early stage by applying a similar model and critically read and helped redrafting the manuscript.

All authors read and approved the final manuscript.

## Pre-publication history

The pre-publication history for this paper can be accessed here:



## References

[B1] Gabbay J, le May A (2004). Evidence based guidelines or collectively constructed "mindlines"? Ethnographic study of knowledge management in primary care. British Medical Journal.

[B2] Therapy Conferences on (1954). How to evaluate a new drug. American Journal of Medicine.

[B3] Therapy Conferences on (1946). The use of placebos in therapy. New York Journal of Medicine.

[B4] Reilly D, Taylor MA (1993). The evidence profile. The mulitdimensional nature of proof. Complementary Therapies in Medicine.

[B5] Jonas WB (2005). Building an Evidence House: Challenges and Solutions to Research in Complementary and Alternative Medicine. Forschende Komplementärmedizin und Klassische Naturheilkunde.

[B6] Black N (1996). Why we need observational studies to evaluate the effectiveness of health care. British Medical Journal.

[B7] Kaptchuk TJ (2001). The double-blind randomized controlled trial: Gold standard or golden calf?. Journal of Clinical Epidemiology.

[B8] Kaptchuk TJ (1998). Powerful placebo: the dark side of the randomised controlled trial. Lancet.

[B9] Kaptchuk TJ (1998). Intentional ignorance: A history of blind assessment and placebo controls in medicine. Bulletin of the History of Medicine.

[B10] Lefering R, Neugebauer E, Abel U, Koch A (1998). Problems of randomized controlled trials (RCT) in surgery. Nonrandomized Comparative Clinical Studies.

[B11] Walach H (2001). Das Wirksamkeitsparadox in der Komplementärmedizin. Forschende Komplementärmedizin und Klassische Naturheilkunde.

[B12] Barrett B, Marchand L, Scheder J, Appelbaum D, Chapman M, Jacobs C, Westergaard R, St.Clair N (2000). Bridging the gap between conventional and alternative medicine. Results of a qualitative study of patients and providers. Journal of Family Practice.

[B13] Moore J, Phipps K, Lewith G, Marcer D (1985). Why do people seek treatment by alternative therapies?. British Medical Journal.

[B14] Lewith GT, Bensoussan A (2004). Complementary and alternative medicine - with a difference: Understanding change in the 21st centruy will help us in the CAM debate. Medical Journal of Australia.

[B15] Ezzo J, Lao L, Berman BM, Stux G, Hammerschlag R (2001). Assessing clinical efficacy of acupuncture: What has been learned from systematic reviews of acupuncture?. Clinical Acupuncture Scientific Basis.

[B16] Ezzo J, Berman B, Hadhazy VA, Jadad AR, Lao L, Singh BB (2000). Is acupuncture effective for the treatment of chronic pain? A systematic review. Pain.

[B17] Linde K, Scholz M, Ramirez G, Clausius N, Melchart D, Jonas WB (1999). Impact of study quality on outcome in placebo-controlled trials of homeopathy. Journal of Clinical Epidemiology.

[B18] Linde K, Melchart D (1998). Randomized controlled trials of individualized homeopathy: A state-of-the-art review. Journal of Alternative and Complementary Medicine.

[B19] Linde K, Clausius N, Ramirez G, Melchart D, Eitel F, Hedges LV, Jonas WB (1997). Are the clinical effects of homoeopathy placebo effects? A meta-analysis of placebo controlled trials. Lancet.

[B20] Diener HC, Kronfeld K, Boewing G, Lungenhausen M, Maier C, Molsberger A, Tegenthoff M, Trampisch HJ, Zenz M, Meinert R, for the GERAC Migraine Study Group (2006). Efficacy of acupuncture for the prophylaxis of migraine: A multicentre randomised controlled clinical trial. Lancet Neurology.

[B21] Skovlund E (1991). Should we tell trials patients that they might receive placebo?. Lancet.

[B22] Skovlund E, Fyllingen G, Landre H, Nesheim BI (1991). Comparison of postpartum pain treatments using a sequential trial design I: paracetamol versus placebo. European Journal of Clinical Pharmacology.

[B23] Skovlund E, Fyllingen G, Landre H, Nesheim BI (1991). Comparison of postpartum pain treatments using a sequential trial design II: naproxen versus paracetamol. European Journal of Clinical Pharmacology.

[B24] Diener HC (1996). Issues in migraine trial design: a case study. The 311C SymposiumPresentations given in the 311C90 Symposion of the 3rd European Headache Conference, 5-8June 1996, SMargherita die Pula.

[B25] Petrovic P, Kalso E, Petersson KM, Ingvar M (2002). Placebo and opioid analgesia - imaging a shared neuronal network. Science.

[B26] Bergmann JF, Chassany O, Gandiol J, Deblos P, Kanis JA, Segrestaa JM, Caulin C, Dahan R (1994). A randomised clinical trial of the effect of informed consent on the analgesic activity of placebo and naproxen in cancer patients. Clinical Trials and Meta-Analysis.

[B27] Kleijnen J, de Craen AJM, Van Everdingen J, Krol L (1994). Placebo effect in double-blind clinical trials: a review of interactions with medications. Lancet.

[B28] Roberts AH, Kewman DG, Mercier L, Hovell M (1993). The power of nonspecific effects in healing: implications for psychosocial and biological treatments. Clinical Psychology Review.

[B29] Thomas KB (1994). The placebo in general practice. Lancet.

[B30] Thomas KB (1987). General practice consultations: is there any point in being positive?. British Medical Journal.

[B31] Uhlenhuth EH, Rickels K, Fisher S, Park LC, Lipman RS, Mock J (1966). Drug, doctor's verbal attitude and clinic setting in the symptomatic response to pharmacotherapy. Psychopharmacologia.

[B32] Uhlenhuth EH, Canter A, Neustadt JO, Payson HE (1959). The symptomatic relief of anxiety with meprobamate, phenobarbitol and placebo. American Journal of Psychiatry.

[B33] Moerman DE (2000). Cultural variations in the placebo effect: Ulcers, anxiety, and blood pressure. Medical Anthropology Quarterly.

[B34] Moerman DE (1983). General medical effectiveness and human biology: Placebo Effects in the treatment of ulcer disease. Medical Anthropology Quarterly.

[B35] Khan A, Khan S (2003). Placebo in mood disorders: the tail that wags the dog. Current Opinion in Psychiatry.

[B36] Kirsch I (1985). Response expectancy as a determinant of experience and behavior. American Psychologist.

[B37] Walach H, Kirsch I, Lilienfeld SO, Lynn SJ, Lohr JM (2003). Herbal treatments and antidepressant medication: Similar data, divergent conclusions. Science and Pseudoscience in Clinical Psychology.

[B38] Hickok JT, Roscoe JA, Morrow GR (2001). The role of patients' expectation in the development of anticipatory nausea related to chemotherapy for cancer. Journal of Pain and Symptom Management.

[B39] Montgomery GH, Bovbjerg DH (1999). Pre-infusion expectations predict prostreatment nausea during repeated adjuvant cheomtherapy infusios for breast cancer. British Journal of Health Psychology.

[B40] Montgomery GH, Phillips AM, Bovbjerg DH (1999). Expectations predict anticipatory ausea in breast cancer patients. Annals of Behavioral Medicine.

[B41] Montgomery GH, Tomoyasu N, Bovbjerg DH, Andrykowski MA, Currie VE, Jacobsen PB, Redd WH (1998). Patients' pretreatment expectations of chemotherapy-related nausea are an independent predictor of anticipatory nausea. Annals of Behavioral Medicine.

[B42] Roscoe JA, Hickock JT, Morrow GR (2000). Patient expectations as predictor of chemotherapy-induced nausea. Annals of Behavioral Medicine.

[B43] Cherkin DC, Eisenberg D, Sherman KJ, Barlow W, Kaptchuk TJ, Street J, Deyo RA (2001). Randomized trial comparing traditional Chinese medical acupuncture, therapeutic massage, and self-care education for chronic low back pain. Archives of Internal Medicine.

[B44] Kalauokalani D, Cherkin DC, Sherman KJ, Koepsell TD, Deyo RA (2001). Lessons from a trial of acupuncture and massage for low back pain. Spine.

[B45] Feinstein AR, Abel U, Koch A (1998). Problems of randomized trials. Nonrandomized Comparative Clinical Studies.

[B46] von Rohr E, Pampallona S, van Wegberg B, Hürny C, Bernhard J, Heusser P, Cerny T (2000). Experiences in the realisation of a research project on anthroposophical medicine in patients with advanced cancer. Schweizer medizinische Wochenschrift.

[B47] Wragg JA, Robinson EJ, Lilford RJ (2000). Information presentation and decision to enter clinical trials: a hypothetical trial of hormone replacement therapy. Social Science and Medicine.

[B48] Pincus T (1997). Analyzing long-term outcomes of clincial care without randomized controlled clinical trials: The Consecutive patient Questionnaire Database. AdvancesThe Journal of Mind-Body Health.

[B49] Lloyd-Williams F, Mair F, Shiels C, Goldstein P, Beaton S, Capewell S, Lye M, Mcdonald R, Roberts C, Connelly D, Hanratty (2003). Why are patients in clinical trials of heart failure not like those we see in everyday practice?. Journal of Clinical Epidemiology.

[B50] Patten SB (2000). Selection bias in studies of major depression using clinical subjects. Journal of Clinical Epidemiology.

[B51] Netter P, Heck S, Müller HJ (1986). What selection of patients is achieved by requestiong informed consent in placebo controlled drug trials?. Pharmacopsychiatry.

[B52] Dahan R, Caulin C, Figea L, Kanis JA, Caulin F, Segrestaa JM (1986). Does informed consent influence therapeutic outcome? A clinical trial of the hypnotic activity of placebo in patients admitted to hospital. British Medical Journal.

[B53] Llewellyn-Thomas HA, McGreal MJ, Thiel E, Fine S, Erlichman C (1991). Patients' willingness to enter clinical trials: measuring the association with perceived benefit and preference for decision participation. Social Science and Medicine.

[B54] Howard KI, Cox WM, Saunders SM, Onken LS, Blaine JD (1990). Attrition in substance abuse comparative treatment research: The illusion of randomization. Psychotherapy and Counseling in the Treatment of Drug Abuse.

[B55] Peto R, Collins R, Gray R (1995). Large-scale randomized evidence: large simple trials and overview of trials. Journal of Clinical Epidemiology.

[B56] Furukawa TA, Streiner DL, Hori S (2000). Discrepancies among megatrials. Journal of Clinical Epidemiology.

[B57] LeLorier J, Gregoire G, Benhaddad A, Lapierre J, Derderian F (1997). Discrepancies between meta-analyses and subsequent large randomized controlled trials. New England Journal of Medicine.

[B58] Shadish WRJ, Cokk TD, Leviton LC (1991). Foundations of Program Evaluation. Theories of Practice.

[B59] Chen HT, Rossi PH (1983). Evaluating with sense. The theory-driven approach. Evaluation Review.

[B60] Cook TD, Wittmann WW (1998). Lessons learned about evaluation in the United States and some possible implications for Europe. European Journal of Psychological Assessment.

[B61] Rossi PH, Freeman HE (1982). Evaluation. A systematic Approach (2nd ed.).

[B62] Wittmann WW, Walach H, Lewith G, Jonas WB, Walach H (2002). Evaluating complementary medicine: Lessons to be learned from evaluation research. Clinical Research in Complementary Therapies: Principles, Problems, and Solutions.

[B63] Mant D (1999). Can randomised trials inform clinical decisions about individual patients?. Lancet.

[B64] Raskin I, Maklan C (1991). Medical treatment effectiveness research. A view from inside the Agency for Health Care Policy and Research. Evaluation and the Health Profession.

[B65] Office USGA (1992). Cross Design Synthesis. A New Strategy for Medical Effectiveness Research.

[B66] Benson K, Hartz AJ (2000). A comparison of observational studies and randomized controlled trials. New England Journal of Medicine.

[B67] Concato J, Shah N, Horwitz RI (2000). Randomized, controlled trials, observational studies, and the hierarchy of research designs. New England Journal of Medicine.

[B68] MacLehose RR, Reeves BC, Harvey IM, Sheldon TA, Russell IT, Black AMS (2000). A systematic review of comparisons of effect sizes derived from randomised and non-randomised studies. Health Technology Assessment.

[B69] Deeks JJ, Dinnes J, Sowden AJ, Sakarovitch C, Song F, Petticrew M, Altman DG, D'Amico (2003). Evaluating non-randomised intervention studies. Health Technology Assessment.

[B70] Walach H, Bösch H, Haraldsson E, Marx A, Tomasson H, Wiesendanger H, Lewith G (2002). Efficacy of distant healing - a proposal for a four-armed randomized study (EUHEALS). Forschende Komplementärmedizin und Klassische Naturheilkunde.

[B71] Güthlin C, Lange O, Walach H (2004). Measuring the effects of acupuncture and homoeopathy in general practice: An uncontrolled prospective documentation approach. BMC Public Health.

[B72] Verhoef MJ, Lewith G, Ritenbaugh C, Thomas K, Boon H, Fonnebo V (2004). Whole systems research: moving forward. Focus on Alternative and Complementary Therapies.

[B73] Medizinprodukte BA (2000). Bekanntmachung über die Registrierung , Zulassung  und Nachzulassung von Arzneimitteln: Abwehr von Arzneimittelrisiken, Anhärung, Stufe II: Johanniskrauthaltige (Hypericum) Humanarzneimittel zur innerlichen Anwendung vom 24.März 2000. Bundesanzeiger.

